# Gastrocolic Fistula: A Shortcut through the Gut

**DOI:** 10.1155/2016/6379425

**Published:** 2016-03-29

**Authors:** Nauzer Forbes, Raed Al-Dabbagh, Peter Lovrics, David Morgan

**Affiliations:** ^1^Division of Gastroenterology, University of Calgary, Calgary, AB, Canada T2N 4Z6; ^2^Division of Gastroenterology, McMaster University, Hamilton, ON, Canada L8S 4K1; ^3^Division of Surgery, McMaster University, Hamilton, ON, Canada L8N 4A6

## Abstract

Gastrocolic fistulas are observed in association with several conditions. Traditionally, peptic ulcer disease was commonly implicated in the formation of gastrocolic fistulas; however, this is now a rare etiology. Here, we present a case of gastrocolic fistula secondary to peptic ulcer disease alone, in addition to reviewing the literature and providing options for diagnosis and treatment.

## 1. Case Presentation

A 47-year-old man presented with a three-month history of anorexia, nausea, vomiting, diarrhea, and weight loss of 30 kg. His bowel movements occurred 12–15 times daily, with abdominal cramps but no gastrointestinal bleeding. No infectious, extraintestinal, or other constitutional symptoms were present. His past medical history included remote peptic ulcer disease (PUD) diagnosed 15 years earlier, for which he was on no current medical therapy, untreated hepatitis C, and intravenous drug use. His sole medication was methadone. The patient's in-hospital course was complicated by pneumonia and sepsis requiring intubation, from which he eventually recovered. Upon recovery from his critical illness, his gastrointestinal symptoms and failure to thrive persisted despite high-calorie enteral feeds via nasogastric tube. The gastroenterology team was involved at this point. Vital signs were stable; physical examination revealed cachexia, minimal shoulder and thigh muscle bulk, thinning hair, fissured nails, and bilateral pitting peripheral edema. Abdominal exam revealed a sunken abdomen and visible ribs, with bulging flanks and a positive fluid level.

**Figure 1:**
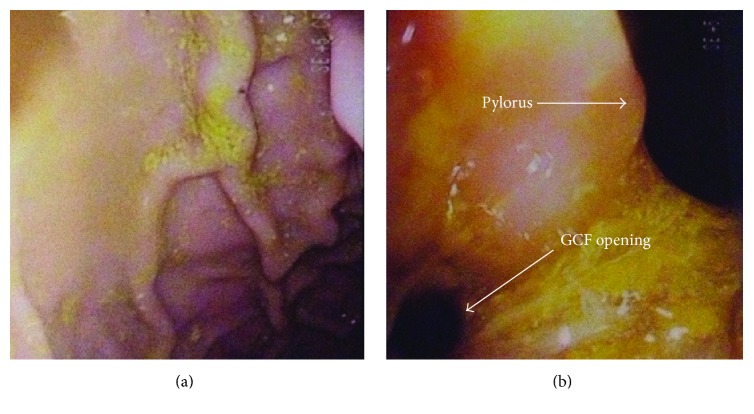
(a) Gastroscopy showing normal gastric rugae with no erythema or ulceration. (b) Gastroscopy showing the opening to the GCF adjacent to the pylorus.

**Figure 2:**
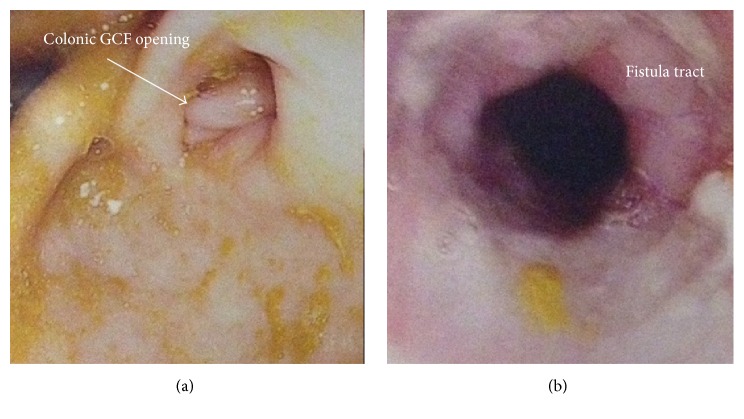
(a) Colonoscopy showing the colonic opening of the GCF. (b) Colonoscopy showing the lumen of the fistula tract.

Laboratory investigations revealed serum potassium of 1.9 mmol/L (normal 3.5–5.0 mmol/L), bicarbonate of 41 mmol/L (normal 22–29 mmol/L), albumin of 9 g/L (normal 35–50 g/L), and creatinine of 35 *μ*mol/L (normal 64–111 *μ*mol/L), along with other markers of malnutrition and hypovitaminosis. Serum and stool workups for infectious causes of diarrhea and celiac disease were negative. The patient underwent gastroscopy and colonoscopy. On gastroscopy, a superfluous lumen was identified proximal to the pylorus (Figure 1(b)), which could not be probed. No surrounding erythema or ulceration was noted (Figure 1(a)). On colonoscopy, a structure with a luminal appearance was identified proximal to the splenic flexure (Figure 2(a)). Upon advancing the colonoscope through this structure inadvertently (Figure 2(b)), gastric rugae were ultimately identified, which suggested the presence of a gastrocolic fistula (GCF). No features suggestive of inflammatory bowel disease (IBD) were identified on endoscopy.

**Figure 3:**
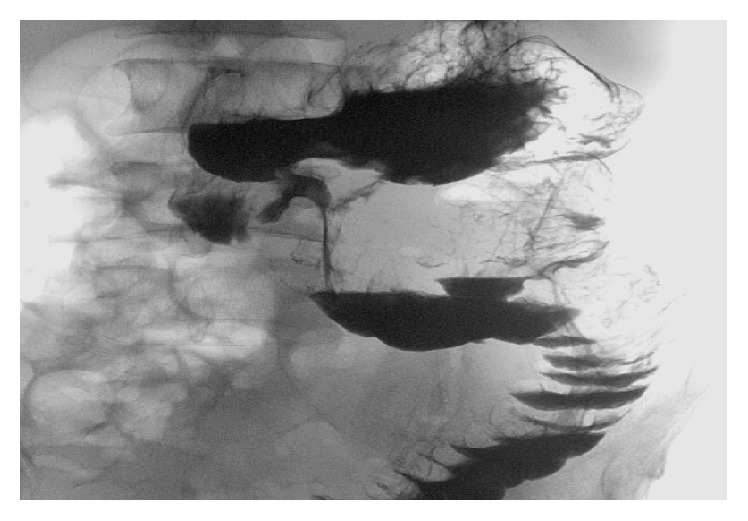
Barium X-ray confirming the GCF.

A barium study revealed immediate filling of the fistula tract from the stomach into the transverse colon (Figure 3). Computed tomography confirmed the results. The patient subsequently received parenteral nutrition and underwent distal gastrectomy and resection of the transverse colon with primary anastomosis. His postoperative course was complicated by bleeding from the colonic anastomosis. He underwent repeat laparotomy; hemostasis was secured and an end colostomy was performed. The patient recovered fully. Final pathology revealed acute and chronic inflammation with ulceration, without features of malignancy or IBD. Repeat endoscopic evaluation 6 months after surgery revealed healthy anastomoses. Successful closure of his colostomy was subsequently performed.

## 2. Discussion

Gastrocolic fistula (GCF) was first described in 1755 as a complication of gastric cancer [1]. It has since been observed in association with several processes, including malignancy, IBD, and pancreatitis, and has also been reported from iatrogenic causes such as percutaneous gastrostomy tube migration or surgical complication [2]. The majority of GCFs in North America result from adenocarcinoma of the colon, while, in Eastern countries, gastric adenocarcinoma is most commonly implicated. The reported incidence is low, at 0.2% [3]. PUD has traditionally been implicated in GCF formation but has now become a rare cause as a result of improved PUD detection and treatment [4].

Typical GCF symptoms include malnutrition, abdominal pain, nausea, vomiting (which can be feculent), pronounced weight loss, and diarrhea. Depending on the cause, several other less specific symptoms are also possible [2]. The most reliable method of diagnosing GCF is via barium enema, which confirms the diagnosis in 90–100% of cases [4]. Endoscopy can diagnose GCF but has a relatively higher miss rate. Computed tomography can be diagnostic, but its primary value lies in staging and preoperative planning, especially in malignant cases.

Several treatment modalities exist, the most widely used of which is surgical resection of the fistula. En-bloc resections and Roux-en-y gastrojejunostomy for GCF have both been described [5]. An over-the-clip method is gaining favour, as it is less invasive and can be offered to patients with higher perioperative risk [6]. GCF closure with human fibrin sealant via gastroscopic approach has also been described [7]. Pharmacological treatment with proton pump inhibitor (PPI) and hydrogen receptor antagonist (H_2_RA) therapy has also been described [8] but has not recently been reported.
